# 4-Hydroxy-17-methylincisterol from *Agaricus blazei* Decreased Cytokine Production and Cell Proliferation in Human Peripheral Blood Mononuclear Cells via Inhibition of NF-AT and NF-**κ**B Activation

**DOI:** 10.1155/2013/435916

**Published:** 2013-02-27

**Authors:** Wei-Jern Tsai, Shih-Chien Yang, Yu-Ling Huang, Chien-Chih Chen, Kai-An Chuang, Yuh-Chi Kuo

**Affiliations:** ^1^National Research Institute of Chinese Medicine, No. 155-1, Section 2, Li-Nung Street, Shih-Pai 112, Taipei, Taiwan; ^2^LS212, Laboratory of Molecular Pharmacology, Department of Life Science, Fu-Jen Catholic University, No. 510, Chung-Cheng Road, Hsinchuang 242, New Taipei, Taiwan; ^3^Graduate School of Biotechnology, Hungkuang University, No. 34, Chung-Chie Road, Sha Lu 443, Taichung, Taiwan; ^4^Department of Biotechnology and Laboratory Science in Medicine, School of Biomedical Science and Engineering, National Yang-Ming University, No. 155, Section 2, Li-Nung Street, Shih-Pai 112, Taipei, Taiwan

## Abstract

*Agaricus blazei* Murill is an edible and medicinal mushroom. In the previous study, we have proved that extracts of *A. blazei* inhibit human peripheral blood mononuclear cell (PBMC) proliferation activated with phytohemagglutinin (PHA). Currently, we purified 4-hydroxy-17-methylincisterol (*4-HM*; C_21_H_33_O_3_) from *A. blazei* investigated its regulatory effects on cytokine productions and cell proliferation of PBMC induced by PHA. The results indicated that *4-HM* suppressed, in activated PBMC, the production and mRNA expression of interleukin-2 (IL-2), IL-4, tumor necrosis factor-**α**, and interferon-**γ** in a concentration-dependent manner. This inhibition was not related to cell viability. While *4-HM* did not affect ERK phosphorylation and its downstream *c-fos* gene expression in PBMC induced by PHA, it decreased both NF-AT and NF-**κ**B activation. The upstream signaling of NF-AT and NF-**κ**B, intracellular calcium concentrations ([Ca^2+^]_i_), and protein kinase C theta (PKC **θ**) activation in PHA-treated PBMC were reduced by *4-HM*. The data demonstrated that the suppressant effects of *4-HM* on cell proliferation in PBMC activated by PHA appeared to be mediated, at least in part, through inhibition of Ca^2+^ mobilization and PKC **θ** activation, NF-AT and NF-**κ**B activation, and cytokine transcripts and productions of PBMC. We suggested that *A. blazei* contained a potential immunomodulator *4-HM*.

## 1. Introduction

*Agaricus blazei *Murill belongs to Basidiomycete and is used as an alternative medicine in Brazil for a long time [1]. Its putative active functions include (a) treatment of ailments, (b) relief atopic dermatitis, and (c) reduction of glycosuria [2, 3]. Although various kinds of healthy foods contain *A. blazei* or its extracts, action mechanisms of *A. blazei *are far beyond complete understanding. The most anticipated pharmacological effect of *A. blazei* is that a proteoglucan (FIII-2-b) isolated from it has antitumor activity through modulation of the natural killer cell activity and macrophage activation [4, 5]. Linoleic acid isolated form *A. blazei *is a bactericidal agent [6]. Steroids identified from *A. blazei* are cytotoxic against HeLa cells [7]. In the previous study, we have proved that ethanolic extracts from* A. blazei *inhibit cytokine productions and cell proliferation in human peripheral blood mononuclear cells (PBMC) induced by phytohemagglutinin (PHA) [8]. PHA is a mitogen for T lymphocytes. It binds to N-acetylgalactosamine glycoproteins expressed on the surface of T cells then activates the cells to produce cytokines and proliferate [9]. Thus, in the present study, the bioactive component was purified from *A. blazei* ethanolic extracts and evaluated in immune response assays. 

Atopic dermatitis is a T-cell-mediated disease and characterized with strong inflammatory cytokines production such as tumor necrosis factor-*α* (TNF-*α*) [10]. One of the therapeutic objectives in tissue inflammation is to reduce the local inflammatory response through the reduction of inflammatory cell activation and proliferation and inflammatory cytokine production. Blockade of the T lymphocyte activation and proliferation and cytokine production is one such anti-inflammatory means [11]. T lymphocyte activation and proliferation are a highly regulated process involving ordered expression of a series of control genes including interleukin-2 (IL-2), IL-4, and interferon-*γ* (IFN-*γ*) [12]. Following interaction with an antigen or a mitogen, a cascade of intracellular signal transduction pathways involving protein kinase C theta (PKC *θ*) activation, calcium mobilization, and translocation of nuclear factor of activated T cells (NF-AT) and nuclear factor *κ*B (NF-*κ*B) is triggered in T lymphocytes, which leads to activation and transcription of a variety of cytokines gene such as IL-2 and IFN-*γ* [13, 14]. 

PKC *θ* is a key enzyme in mediating T lymphocyte activation and survival and regulates the activation of NF-*κ*B and NF-AT, which are included in cytokine gene expression such as IL-2, IL-4, and IFN-*γ* [15]. Calcium is a major cellular second messenger and the decreasing of intracellular calcium concentration ([Ca^2+^]_i_) would induce impairment of cytokine productions in T lymphocytes [12, 16]. One of the downstream effects of [Ca^2+^]_i_ increase is regulation of NF-AT dephosphorylation and its subsequent nuclear translocation [13]. NF-AT is required for activation and proliferation of T lymphocytes [13]. The specific NF-AT inhibitor, cyclosporin A (CsA), blocks cell proliferation and IL-2 productions in T lymphocytes induced by PHA [17]. The transcription factor NF-*κ*B has been shown to participate in cell proliferation and carcinogenesis [14, 18]. It regulates several proinflammatory cytokine productions such as IL-1*β* and TNF-*α* and promotes chronic inflammatory diseases including atherosclerosis and type 2 diabetes [19]. By coordinated binding of NF-*κ*B and NF-AT, the induction of a defined genetic program is initiated that leads to the production of cytokines such as IL-2, IL-4, TNF-*α*, and IFN-*γ* [20, 21]. Concurrently, a number of recruitment and assembling of phosphorylated-protein complexes cause signal transduction of extracellular signal-regulated kinase (ERK), which is involved in regulation of cytokines gene expression in T lymphocytes [22, 23]. The activation of ERK is involved in T-cell proliferation [24]. ERK activation affects *c-fos* expression, and both ERK and *c-fos* are involved in cytokines gene expression and T-cell growth [25]. Furthermore, Growth modulators or other external events that affect cytokine gene expression and cells proliferation in the T cell are ultimately likely to act by controlling the expression or function of these signals [12, 26].

 In the present study, 4-hydroxy-17-methylincisterol (*4-HM*) was purified from ethanolic extracts of *A. blazei*, PHA-stimulated PBMC were used as target cells, and effects of *4-HM* on immune responses in PBMCs induced by PHA were evaluated. We determined the actions of *4-HM* on production and gene expression of IL-2, IL-4, TNF-*α* and IFN-*γ*, Ca^2+^ mobilization, and activation of NF-AT, NF-*κ*B, ERK, and PKC *θ* in PBMC induced by PHA and examined their roles in regulation of PBMC proliferation. 

## 2. Materials and Methods

### 2.1. The Source of *A. blazei*

*A. blazei* mushrooms were purchased from Chinese medicine shops in Taipei and identified by Dr. Y.-L. Huang and Professor C.-C. Chen, resident medicinal-fungus experts of the National Research Institute of Chinese Medicine and Graduate School of Biotechnology, Hungkuang University, respectively.

### 2.2. **** *4-HM* Purified from *A. blazei*

**Figure 1:**
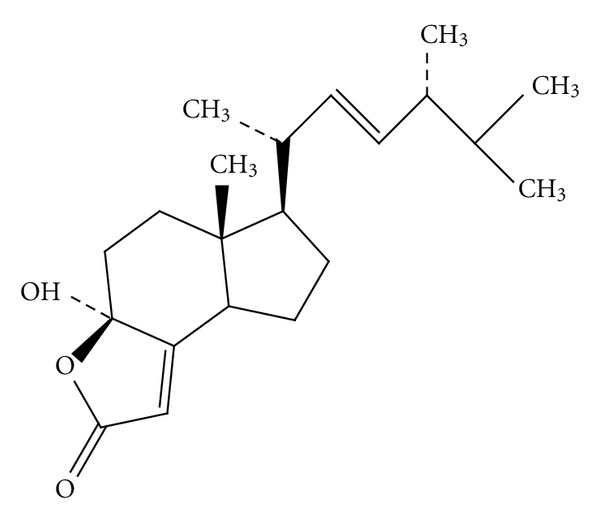
The structure of *4-HM. *

5 Kg of dried *A. blazei *were ground and extracted with ethanol (EtOH) three times (5 L × 3). The ethanolic extracts were evaporated with Rotavapor under vacuum. The crude extracts were partitioned successively between ethyl acetate (EtOAc) followed by butanol (BuOH) and methanol (MeOH), respectively. The bioactive EtOAc fraction was subjected to silica gel chromatography (sample/adsorbent (v/v) = 1/8). Extensive gradient elution was then employed using n-hexane, EtOAc, dichloromethane (CH_2_Cl_2_), acetone, and MeOH, respectively, to yield 23 fractions. The like fractions were combined to give 12 main fractions with monitoring by thin-layer chromatography and the solvent was removed under reduced pressure. Each combined fraction was further purified by rechromatography and recrystallization.* 4-HM* (23 mg; C_21_H_33_O_3_; M.W. 333; Figure 1) was isolated from fraction 8. The nuclear magnetic resonance and mass spectral data for *4-HM* were identical with the previously reported by Togashi et al. [27]. The purity of *4-HM* is above 98%. *4-HM* was dissolved by dimethylsulfoxide (DMSO) to a concentration of 100 mM and stored at 4°C until for use.

### 2.3. Human Subjects

Twenty healthy male subjects (22 to 35 yr, mean age 26 yr) were chosen for this investigation. The experimental protocol had been reviewed and approved by the Human Experimentation Committee of Fu-Jen Catholic University (Approved number: C9819). Written informed consent was obtained from each and every subject.

### 2.4. Preparation of PBMC

 Heparinized human peripheral blood samples (35 mL) were obtained from healthy donors. PBMC was isolated by the Ficoll-Paque (specific gravity 1.077) gradient density method as described previously [28]. The peripheral blood was centrifuged to remove the plasma and blood cells were diluted with phosphate-buffered saline (PBS; pH 7.2) then centrifuged in a Ficoll-Paque discontinuous gradient at 420 ×g for 30 min. The PBMC layer was collected and washed with cold distilled water and 10x Hanks' buffer saline solution (HBSS) to remove red blood cells. The cells were resuspended to a concentration of 2 × 10^6^ cells/mL in Rosewell Park Memorial Institute Medium (RPMI)-1640 medium supplemented with 2% fetal calf serum (FCS), 100 U/mL penicillin, and 100 *μ*g/mL streptomycin.

### 2.5. Determination of Cytokine Production in PBMC

 PBMC (2 × 10^5^ cells/well) were cultured with PHA (5 *μ*g/mL) alone, or in combination with varying concentrations of *4-HM* for 3 days. The cell supernatants were then collected and assayed for IL-2, IL-4, IFN-*γ*, and TNF-*α* concentrations by enzyme-linked immunosorbent assay (ELISA; R&D systems, Minneapolis, MN, USA). 

### 2.6. Extraction of Total Cellular RNA

The protocols followed the previous published report [16]. PBMCs (5 × 10^6^) were activated with or without PHA (5 *μ*g/mL) and cocultured with *4-HM* (2.5, 5, 10 *μ*M). PBMCs were collected and lysed by RNA-Bee (Tel-Test Inc., Friendswood, TX, USA). After centrifugation, the supernatants were extracted with a phenol-chloroform mixture. The extracted RNA was precipitated with 100% cold ethanol. The total cellular RNA was pelleted by centrifugation and redissolved in diethyl pyrocarbonate (DEPC-) treated H_2_O. The concentration of RNA was calculated by measuring the optical density at 260 nm. 

### 2.7. Reverse Transcription-Polymerase Chain Reaction (RT-PCR)

The RT-PCR was performed by a method described previously [29]. Aliquots of 1 *μ*g of RNA were reverse transcribed to cDNA using the Advantage RT-for-PCR kit from CLONTECH according to the manufacturer's instructions. Briefly, 10 *μ*L of cDNA was mixed with 0.75 *μ*M primers, 4 units of Taq polymerase, 10 *μ*L of reaction buffer (2 mM Tris-HCl, pH 8.0; 0.01 mM ethylenediaminetetraacetate, EDTA; 0.1 mM dithiothreitol, DTT; 0.1% Triton X-100; 5% glycerol; and 1.5 mM MgCl_2_), and 25 *μ*L of water in a total volume of 50 *μ*L. As shown in Table 1, all primer pairs for the *c-fos*, IL-2, IFN-*γ*, IL-4, and TNF-*α* were designed from the published human cDNA sequence data [30–33]. The glyceraldehyde-3-phosphate dehydrogenase (GAPDH) mRNA as internal control. The PCR was done by the following setting of the air thermocycler: denaturing temperature of 94°C for 1 min, annealing temperature of 60°C for 1 min, and elongation temperature of 72°C for 80 sec for the first 35 cycles and finally elongation temperature of 72°C for 10 min. Following the reaction, the amplified products were taken out of the tubes and run on 2% agarose gel.

### 2.8. Real-Time PCR

The real-time PCR was performed by TaqMan PCR assay using an ABI prism 7700 sequence detection system (Applied Biosystems, Foster City, CA, USA). The reaction conditions were 50°C for 2 min following by 10 min at 95°C and 40 cycles of 15 sec at 95°C and 1 min at 60°C. The relative cytokines mRNA levels were indicated as % referred to DMSO + PHA control.

### 2.9. Determination of [Ca^2+^]_i_ in PBMC

PBMC were loaded with 5 *μ*M fluo-3-acetoxymethyl ester (Fluo-3-AM) at 37°C for 30 min. The cells were then resuspended in Ca^2+^-free RPMI-1640 medium without phenol red to a concentration of 4 × 10^6^ cells/mL. In each experiment, 0.5 mL of cells suspension was equilibrated with an equal volume of 2 mM Ca^2+^-containing medium at 37°C. The cells were added with 2.5 *μ*L of 0.1% DMSO or *4-HM* (2.5, 5, 10 *μ*M) at the 40th sec then stimulated with 2.5 *μ*L of PHA (5 *μ*g/mL) and the changes in fluorescence with time were recorded. During the measurement, the cells' suspension was kept at 37°C and continuously stirred. The fluorescent activity was recorded at Ex = 340, 380 nm and Em = 505 nm using an F-4500 fluorescence spectrophotometer (Hitachi, Tokyo, Japan) with a multiwavelength time-scan program.

### 2.10. Luciferase Assay

Jurkat cells (5 × 10^4^), a T leukemia cell line, were stably transfected with pGL4.30 (luc2P/NFAT-RE/Hygro or luc2P/NF*κ*B-RE/Hygro), respectively, seeded into 96-well plates and cultured with PHA (5 *μ*g/mL) in the presence or absence of *4-HM* (2.5, 5, 10 *μ*M), cyclosporine A (CsA; 2 *μ*M), or pyrrolidine dithiocarbamate (PDTC; 25 *μ*M) for 4 hr. Total cell lysates were extracted with 1X reporter lysis buffer (Promega, USA), then 10 *μ*g of total cell lysates were used to determine luciferase activity by the Luciferase Assay System (Promega, USA).

### 2.11. Western Blot Analysis

The cytosolic and membrane proteins were extracted from PBMC by a method described previously [17]. PBMC (1 × 10^7^ cells) were applied into each well of a 6-well flat-bottomed plate and treated with PHA (5 *μ*g/mL) in the presence or absence of *4-HM* (2.5, 5, and 10 *μ*M). The plates were incubated in 5% CO_2_-air humidified atmosphere at 37°C for various times. Cells were harvested and suspended in lysis buffer (10 mM Tris, 150 mM NaCl, 2 mM EDTA, 2 mM EGTA, 1 mM Na_3_VO_4_, 0.2 mM PMSF, 50 *μ*M NaF, 2 *μ*M leupeptin, and 0.7 *μ*g/mL pepstatin A) with 0.1% Triton X-100 for 10 min. The cytosolic fraction was collected by centrifugation at 10,000 ×g for 3 min. Pellets were resuspended in lysis buffer with 0.5% Triton X-100 for 60 min. The membrane fraction was collected from supernatant by centrifugation at 10,000 ×g for 20 min. The 30 *μ*g proteins were dissolved in the dissociation buffer (2% SDS, 5%  *β*-mercaptoethanol, 0.05 M Tris-HCl, and 20% glycerol, pH 7.6) and boiled for 5 min. Then proteins were resolved by 10% SDS-PAGE and transferred to nitrocellulose filters. After blocking the filters with a solution containing 1% bovine serum albumin (BSA), the filters were incubated with mouse monoclonal Abs raised against human ERK, pERK, PKC *θ*, cyclins D3, E, A, or B (BD Biosciences, San Diego, CA, USA). Specific reactive proteins were detected by an enhanced chemiluminescence method, employing a rabbit antimouse Ig Ab linked to horseradish peroxidase (Pierce, Rockford, IL, USA).

### 2.12. Lymphoproliferation Test

 The lymphoproliferation test was modified from that previously described in [29]. The DNA synthesis in proliferating cells was labeled with ^3^H-thymidine. The density of PBMC was adjusted to 2 × 10^6^ cells/mL before use. 100 *μ*L of cell suspension was applied into each well of a 96-well flat-bottomed plate (Nunc 167008, Nunclon, Raskilde, Denmark) with or without 5 *μ*g/mL PHA. *4-HM* (2.5, 5, 10 *μ*M) or various pharmacological inhibitors including CsA; (2 *μ*M) and PDTC (25 *μ*M) were added to the cells. The plates were incubated in 5% CO_2_-air humidified atmosphere at 37°C for 3 days. Subsequently, ^3^H-thymidine (1 *μ*Ci/well, NEN) was added into each well. After a 16-hr incubation, the cells were harvested on glass fiber filters by an automatic harvester (Dynatech, Multimash 2000, Billingshurst, UK). Radioactivity in the filters was measured by liquid scintillation counting. The inhibitory activity of* 4-HM* on PBMC proliferation was calculated by the following formula:

(1) Inhibitory  Activity  (%)  =Control  Group  (CPM)−Experiment  group  (CPM)Control  group  (CPM)   ×100.

### 2.13. Determination of Cell Viability

Approximately 2 × 10^5^ PBMC were cultured with 0.1% DMSO or *4-HM* (2.5, 5, 10 *μ*M) for 4 days. Total, viable, and nonviable cell numbers were counted under the microscope with the help of a hemocytometer following staining by trypan blue and cell viability was calculated.

### 2.14. Reagents, Antibodies, and Kits

Reagents were obtained from Sigma or Merck. Antibodies were purchased from BD Biosciences (San Diego, CA, USA). Rabbit antimouse Ig Ab linked to HRP for detection in the Western blotting was obtained from Pierce Inc. (Rockford, USA). The kits for ELISA of cytokines were from R&D Systems (Minneapolis, USA). RNA-Bee reagent for RNA extraction was obtained from Tel-Test Inc. (Friendswood, USA). The Advantage RT-for-PCR kit was from CLONTECH (Palo Alto, CA, USA). The TaqMan PCR reagents were bought from Applied Biosystems (Foster City, CA, USA).

### 2.15. Statistical Analysis

Data were presented as mean ± SD, and the differences between groups were assessed with Student's *t*-test at a significant level of *P* < 0.05.

## 3. Results

### 3.1. Inhibitory Effects of *4-HM* on Cytokines Production in PBMC

**Figure 2:**
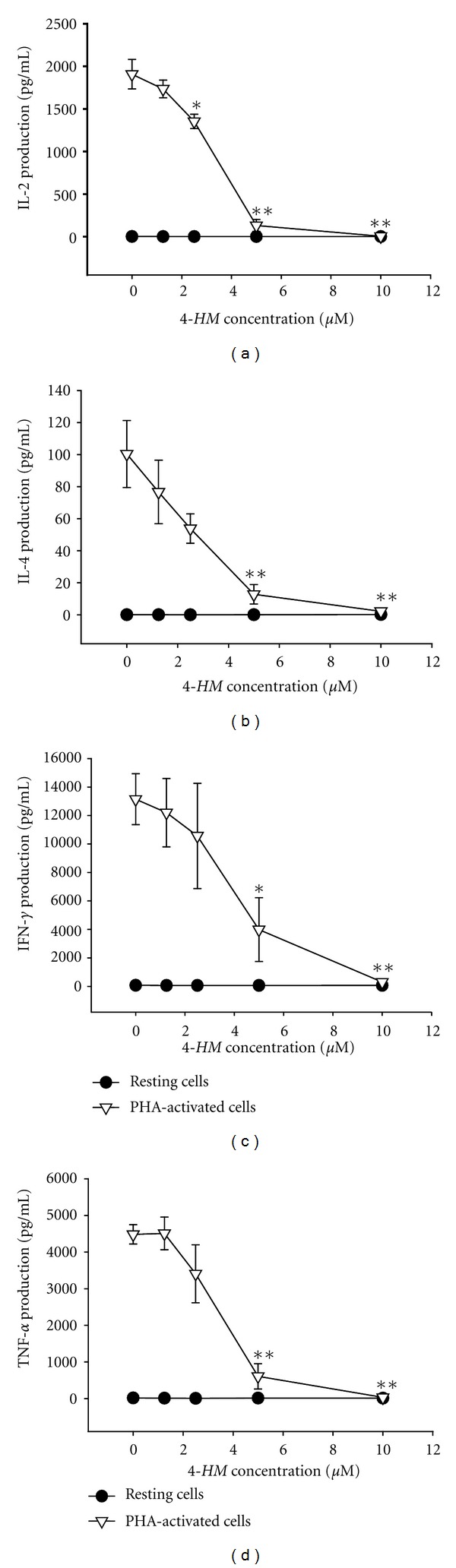
IL-2, IL-4, IFN-*γ*, and TNF-*α* productions in PBMCs treated with *4-HM*. PBMC (2 × 10^5^ cells/well) were treated by 0, 1.25, 2.5, 5, and 10 *μ*M of *4-HM* with or without PHA (5 *μ*g/mL) for 72 hr. Then the cell supernatants were collected and (a) IL-2, (b) IL-4, (c) IFN-*γ*, and  (d) TNF-*α*  concentrations were determined by ELISA. Each point is the mean ± SD of three independent experiments with PBMC from different individuals. *P < 0.05, **P < 0.01: versus PHA control.

**Figure 3:**
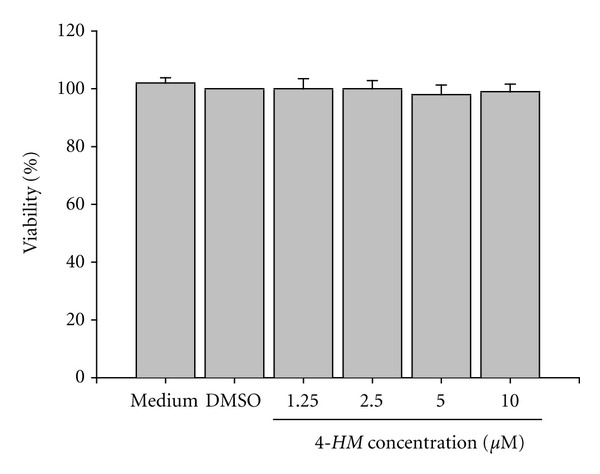
Effects of *4-HM* on PBMC viability. PBMC (2 × 10^5^ cells/well) were treated with medium, 0.1% DMSO, or the indicated concentration of *4-HM* for 4 days. Numbers of total, viable, and nonviable cells were counted after trypan blue staining. Each bar represents the mean ± SD of three independent experiments with PBMC from different individuals.

 To investigate whether *4-HM* affected cytokines production in PBMC, the cells were stimulated with PHA in the presence or absence of varying concentrations of *4-HM* (1.25, 2.5, 5.0, 10 *μ*M) for 3 days. Supernatants were then collected, and the production of IL-2, IL-4, IFN-*γ*, and TNF-*α* were assayed by *ELISA*, respectively. As shown in Figure 2, the stimulated production of IL-2, IL-4, IFN-*γ*, and TNF-*α* in activated PBMC was significantly suppressed by *4-HM *(*P* < 0.05 or *P* < 0.01). Furthermore, the inhibitory activities of *4-HM* were concentration dependent. At 10 *μ*M, the stimulated productions of IL-2, IL-4, IFN-*γ*, and TNF-*α* in activated PBMC were completely blocked by *4-HM*, with their concentrations returning to almost the same as those produced in resting cells. However, the inhibitory effect of *4-HM* on cytokine productions was not related to direct cytotoxicity since the viabilities of PBMC were not significantly decreased following treatment with 1.25, 2.5, 5.0, or 10 *μ*M of *4-HM* for 4 days (Figure 3).

### 3.2. Effects of *4-HM* on IL-2, IL-4, IFN-*γ*, and TNF-*α* mRNA Expressions in PBMC

**Figure 4:**
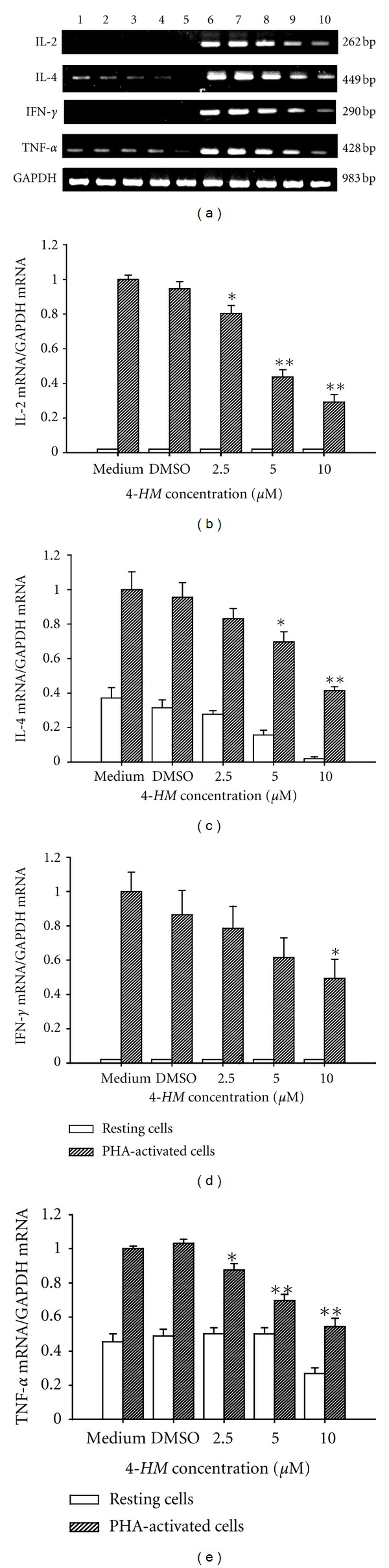
Effects of* 4-HM* on cytokine gene expression in PBMC, as detected with RT-PCR. PBMCs (5 × 10^6^) were cultured with PHA (5 *μ*g/mL) or 2.5, 5, and 10 *μ*M *4-HM* for 18 hr. The total cellular RNA was isolated from PBMC and aliquots of RNA (1 *μ*g) were reverse-transcribed for synthesis of cDNA. PCR was done as described in Section 2. (a) Following the reaction, the amplified products were taken out of the tubes and run on 2% agarose gel. Lane 1: medium, Lane 2: 0.1% DMSO, Lanes 3 to 5: 2.5, 5, or 10 *μ*M *4-HM*, Lane 6: PHA, Lane 7: PHA + 0.1% DMSO, Lanes 8 to 10: PHA + 2.5, 5, or 10 *μ*M *4-HM*. Each band was quantitated using laser scanning densitometer SLR-2D/1D (Biomed Instruments Inc., Fullerton, CA, USA). The ratios of (b) IL-2, (c) IL-4, (d) IFN-*γ*, or (e) TNF-*α*  mRNA to GAPDH mRNA were calculated. Each bar is the mean ± SD of three independent experiments with PBMC from different individuals. *P < 0.05, **P < 0.01: versus DMSO group.

Therefore, we examined whether the cytokine mRNA expressions in activated PBMC were inhibited by *4-HM*. Total cellular RNA was extracted from activated PBMC in the presence or absence of *4-HM* (2.5, 5, and 10 *μ*M) and analyzed by RT-PCR. As shown in Figure 4(a), the levels of IL-2, IL-4, IFN-*γ*, and TNF-*α* mRNA in PBMC were significantly induced by PHA (Lane 6) and DMSO (0.1%) did not affect this induction (Lane 7). By contrast, PCR products for these cytokines amplified from activated PBMC RNA preparations were reduced by *4-HM*. As shown in Figures 4(b) to 4(e), laser densitometry analysis demonstrated that the ratios of IL-2, IL-4, IFN-*γ*, and TNF-*α* to GAPDH mRNAs in PHA-activated PBMC cells were significantly decreased by *4-HM* (*P* < 0.05 or *P* < 0.01). We also utilized real-time PCR to confirm these data. As shown in Table 2, various concentrations of *4-HM* significantly reduced IL-2, IL-4, IFN-*γ*, and TNF-*α* mRNA expressions in PBMC stimulated by PHA. Thus, those data consistent with inhibition of IL-2, IL-4, IFN-*γ*, and TNF-*α* productions by *4-HM*. 

### 3.3. **** *4-HM* Did Not Affect ERK Activation in PBMC Induced by PHA

**Figure 5:**
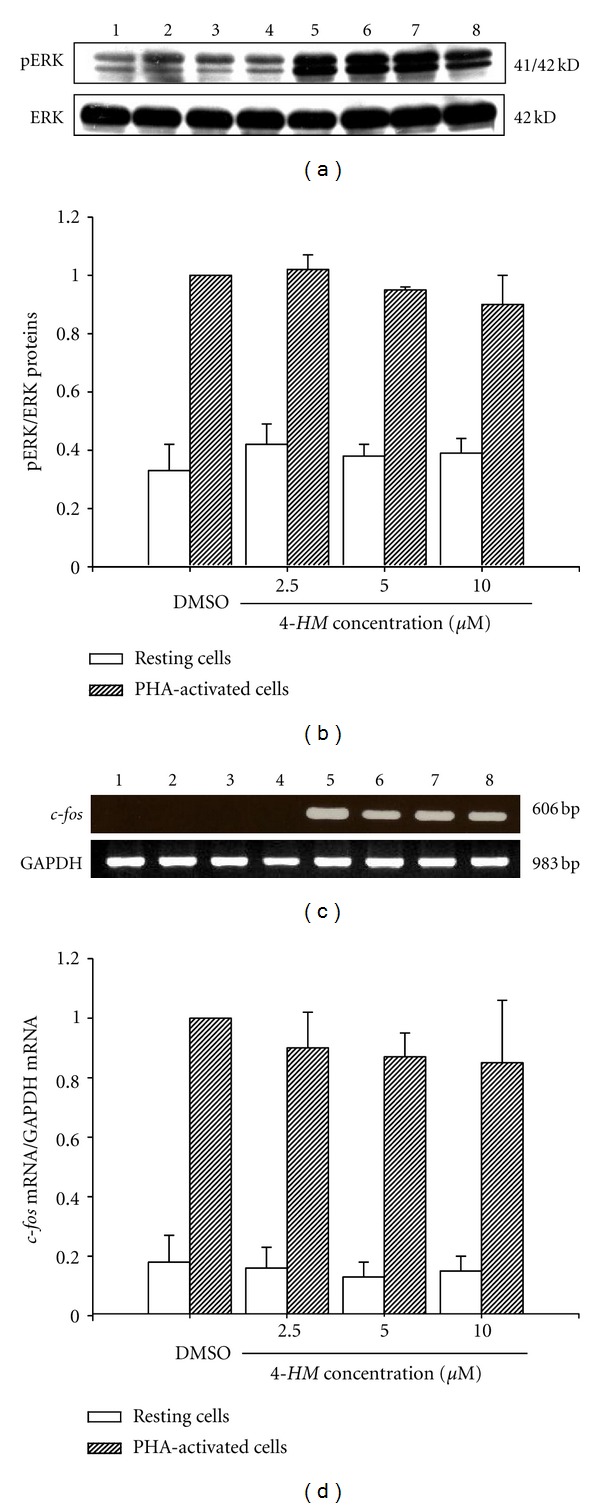
*4-HM* did not affect ERK phosphorylation and *c-fos* gene expression in PBMC induced by PHA. (a) PBMCs (1 × 10^7^ cells) were treated with DMSO (0.1%), or *4-HM* (2.5, 5, 10 *μ*M) in the presence or absence of PHA (5 *μ*g/mL) for 2 hr. Lysates (50 *μ*g/mL) were collected and run on a 10% SDS-PAGE gel and analyzed by immunoblotting with anti-pERK, or ERK Ab. (b) Bar graph indicates the ratio of pERK to ERK proteins. (c) PBMCs (5 × 10^6^) were cultured with PHA (5 *μ*g/mL) or 2.5, 5, and 10 *μ*M *4-HM* for 18 hr. The total cellular RNA was isolated from PBMC and RT-PCR was done as described in Section 2. Following the reaction, the amplified product was run on 2% agarose gel. (d) The ratio of *c-fos* mRNA to GAPDH mRNA was calculated. Each band was quantitated using laser scanning densitometer SLR-2D/1D (Biomed Instruments Inc., Fullerton, CA, USA). Each bar represents mean ± SD from three independent experiments with PBMC from different individuals. Lane 1: 0.1% DMSO, Lanes 2 to 4: 2.5, 5, or 10 *μ*M *4-HM*, Lane 5: PHA + 0.1% DMSO, Lanes 6 to 8: PHA + 2.5, 5, or 10 *μ*M *4-HM*.

To delineate whether *4-HM* reduction of IL-2, IL-4, IFN-*γ*, and TNF-*α* gene expression in PBMC stimulated with PHA was related to ERK signaling, cytosolic proteins were extracted from PBMC and phosphorylation of ERK was determined by Western blotting. As shown in Figure 5(a), DMSO (Lane 1) and *4-HM* (2.5, 5, 10 *μ*M; Lanes 2 to 4) did not affect the levels of ERK proteins. Compared with DMSO control, *4-HM* alone did not influence the phosphorylation of ERK in unstimulated PBMC. After treatment with PHA for 2 hr, the phosphorylation of ERK was detected in PBMC (Lane 5) while the presence of *4-HM* did not affect ERK activation (Lanes 6 to 8). Taken together, these results indicated that *4-HM* did not affect the activation of ERK in PBMC induced by PHA (Figure 5(b)). We suggest that *4-HM* decrease IL-2, IL-4, IFN-*γ*, and TNF-*α* gene expressions in PBMC are not through modulation of ERK pathway. 

### 3.4. **** *4-HM* Did Not Decrease c-fos Gene Expressions in PBMC

To confirm that *4-HM* did not affect ERK phosphorylation, we determined effects of *4-HM* on its downstream gene* c-fos* mRNA expression in PBMC activated with PHA by RT-PCR. As shown in Figure 5(c), PHA significantly induced *c-fos* mRNA expression in PBMC (Lane 5). However, different concentrations of *4-HM* (2.5, 5, 10 *μ*M) did not influence the levels of *c-fos* mRNA in PBMC activated by PHA (Lanes 6, 7, and 8). The laser densitometry analysis demonstrated that compared with vehicle group (0.1% DMSO), the ratio of *c-fos* to GAPDH mRNAs in PHA-activated PBMC was not changed by *4-HM*. Based on these data, we confirm that *4-HM* did not affect the ERK-*c-fos* signaling pathway.

### 3.5. NF-AT and NF-*κ*B Activations Are Inhibited by *4-HM*

**Figure 6:**
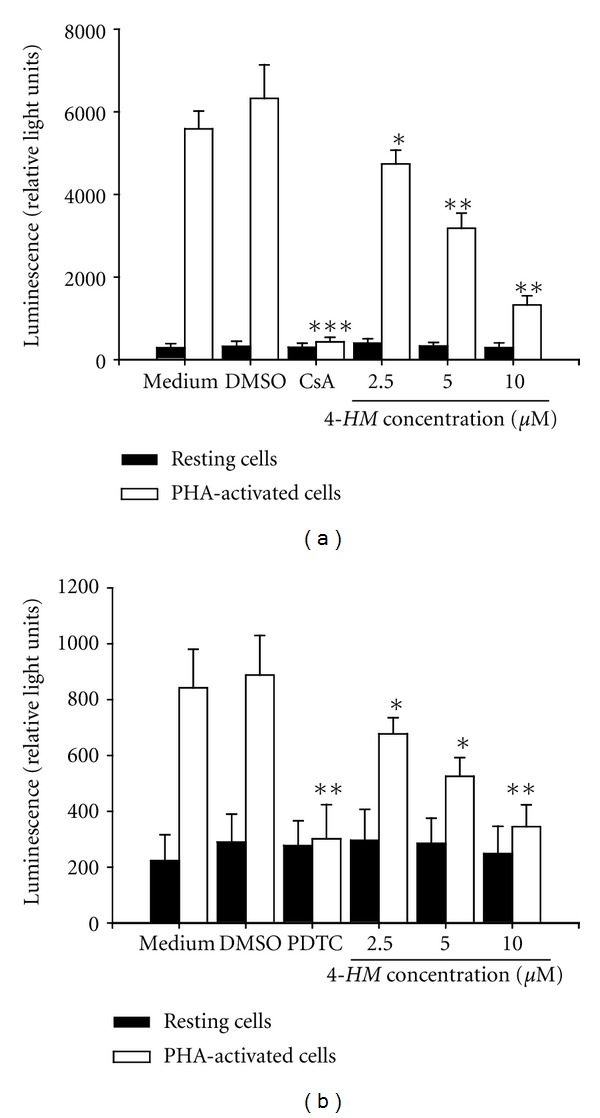
Effects of *4-HM* on NF-AT and NF-*κ*B activation. Jurkat cells (5 × 10^4^) stably transfected with pGL4.30 (luc2P/NFAT-RE/Hygro or luc2P/NF*κ*B-RE/Hygro) were cultured with PHA (5 *μ*g/mL) in the presence or absence of CsA (2 *μ*M), PDTC (20 *μ*M), or *4-HM* (2.5, 5, and 10 *μ*M) for 4 hr. Total cell lysates were extracted with 1x reporter lysis buffer (Promega, USA), then 10 *μ*g of total cell lysates were used to determine luciferase activity by the Luciferase Assay System (Promega, USA). Each bar is the mean ± SD of three independent experiments. *P < 0.05, **P < 0.01, ***P < 0.001: versus PHA + DMSO control.

Attempts to demonstrate whether *4-HM* suppressed IL-2, IL-4, IFN-*γ*, and TNF-*α* mRNA expression was related to the initial transcriptional transactivation event, the effect of *4-HM* on activation of NF-AT and NF-*κ*B was examined by the luciferase reporter assay. As shown in Figures 6(a) and 6(b), PHA induced NF-AT and NF-*κ*B activation in Jurkat cells and DMSO (0.1%) did not affect this activation. NF-AT inhibitor CsA (2 *μ*M) and NF-*κ*B inhibitor PDTC (20 *μ*M) significantly interrupted the luciferase activity, respectively (*P* < 0.001; *P* < 0.01). In the presence of *4-HM*, activation of NF-AT and NF-*κ*B was blocked by *4-HM *in a dose-dependent manner (*P* < 0.05; *P* < 0.01). These results suggested that *4-HM*-reduced cytokine mRNA expression was related to interruption of the activation of NF-AT and NF-*κ*B.

### 3.6. Calcium Mobilization in PBMC Affected by *4-HM*

**Figure 7:**
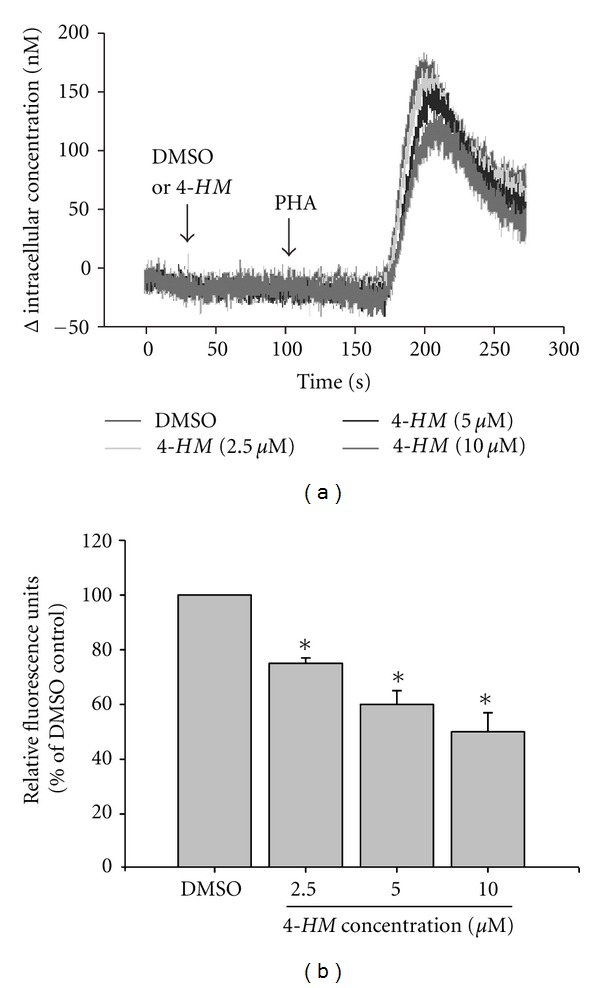
Effects of *4-HM* on Ca^2+^ mobilization in PBMC stimulated with PHA. (a) PBMC were loaded with 1 *μ*M Fluo-3-AM at 37°C for 30 min. The cells were then resuspended in RPMI-1640 medium without phenol red to a concentration of 4 × 10^6^ cells/mL. In each experiment, 0.5 mL of equilibrated PBMC suspension was added with 2.5 *μ*L of DMSO (0.1%) or *4-HM* (2.5, 5, or 10 *μ*M) at the 30th sec then stimulated with 2.5 *μ*L of PHA (5 *μ*g/mL) at the 110th sec and the changes in fluorescence with time were recorded. The fluorescent activity was recorded by an F-4500 fluorescence spectrophotometer (Hitachi, Tokyo, Japan) with multiwavelength time scan program. (b) The graph represents relative fluorescent units (% of DMSO control) of each test and each bar is the mean ± SD of six independent experiments. *P < 0.05: versus DMSO control.

Calcium mobilization is a very early event in PBMC activation that is necessary for activation of NF-AT [34]. To study whether the impairment of NF-AT activation in PBMC was related to Ca^2+^ mobilization, the cells were incubated with or without *4-HM* (2.5, 5, and 10 *μ*M), and [Ca^2+^]_i_  in cells was determined. The results are shown in Figure 7. The kinetics and amplitude of the Ca^2+^ influx were generated in PBMC stimulated by PHA (Figure 7(a)). When PBMCs were pretreated with DMSO, then PHA was added into cells, [Ca^2+^]_i_ began to increase and got to maximal about at the 200th sec. However, the increasing of [Ca^2+^]_i_  in PBMC was decreased by *4-HM* with a dose-dependent manner. As shown in Figure 7(b), the relative fluorescent units of each test were calculated from 6 independent experiments. Compared with DMSO control group, 2.5, 5, and 10 *μ*M of 4-HM significantly reduced [Ca^2+^]_i_ in PBMC induced by PHA (*P* < 0.05). Therefore, inhibition of activation of NF-AT might be explained by a failure to Ca^2+^ mobilization in *4-HM*-treated PBMC.

### 3.7. The Activation of PKC *θ* in PBMC Was Reduced by *4-HM*

**Figure 8:**
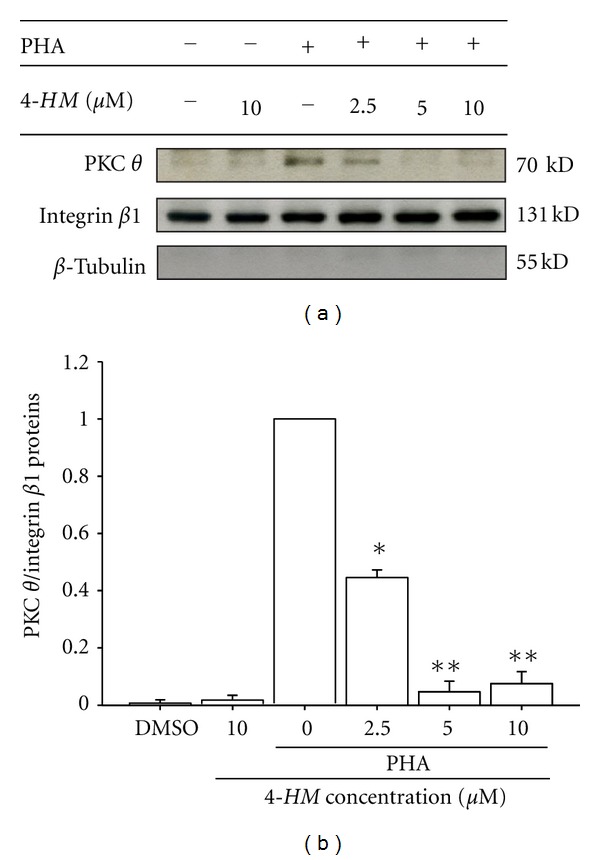
Effects of *4-HM* on PKC *θ* activation in PBMC activated with PHA. PBMC (1.5 × 10^7^ cells) were activated with or without PHA (5 *μ*g/mL) and cocultured with indicated concentrations of *4-HM* (2.5, 5, and 10 *μ*M) for 1 hr. The membrane fraction was extracted and subjected to 10% SDS-PAGE and detected by immunoblotting with anti-PKC *θ*, integrin *β*1, or *β*-tubulin Ab. The ratio of PKC *θ* to integrin *β*1 was shown in bar graph. Each bar represents mean ± SD from three independent experiments. *P < 0.05, **P < 0.01: versus PHA control.

To elucidate whether *4-HM* inhibited NF-*κ*B activation through modulation of PKC *θ* activation, PBMCs were treated with or without *4-HM *(2.5, 5, and 10 *μ*M) for 1 hr and membrane proteins were prepared then analyzed by Western blotting. As shown in Figure 8, membrane fractions expressed membrane control protein integrin *β*1 but did not express cytosolic control *β*-tubulin. Membrane expression of PKC *θ* was significantly stimulated by the treatment of PHA (5 *μ*g/mL) for 1 hr (Lane 3; *P* < 0.05). Various concentrations of *4-HM* decreased the levels of PKC *θ* in membrane protein extracts from PHA-activated PBMC (Lanes 4 to 6). However, *4-HM* did not affect integrin *β*1 expression in resting or activated PBMC. The laser densitometry analysis demonstrated that the ratios of PKC *θ* to integrin *β*1 proteins in PHA-activated PBMC were significantly decreased by the treatment of *4-HM* (*P* < 0.05; *P* < 0.01). On the other hand, we also determined PKC *θ* expression in cytosolic proteins from PBMC. The preliminary data indicated that compared with the unstimulated group (PKC *θ*/*β*-tubulin from densitometry analysis: 0.62), PHA stimulation attenuated PKC *θ* levels (PKC *θ*/*β*-tubulin from densitometry analysis: 0.32) and *4-HM* (10 *μ*M) could restore this level (PKC *θ*/*β*-tubulin from densitometry analysis: 0.58). These results indicated that *4-HM*-inhibited NF-*κ*B activation was related to reduction of PKC *θ* activation.

### 3.8. Effects of *4-HM* on PBMC Proliferation

**Figure 9:**
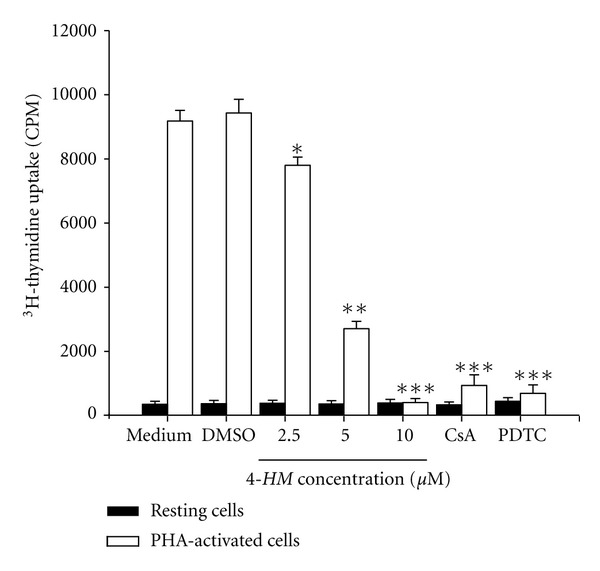
Effects of *4-HM* on PBMC proliferation. PBMC (2 × 10^5^ cells/well) were treated with medium, DMSO (0.1%), indicated concentration of *4-HM*, CsA (2 *μ*M), or PDTC (20 *μ*M) with or without PHA (5 *μ*g/mL) for 3 days. The proliferation of cells was detected by ^3^H-thymidine uptake (1 *μ*Ci/well). After a 16 hr incubation, the cells were harvested by an automatic harvester, then radioactivity was measured by liquid scintillation counting. Each bar represents mean ± SD from three independent experiments with PBMC from different individuals. *P < 0.05, **P < 0.01, ***P < 0.001: versus DMSO control.

Activation of NF-*κ*B and NF-AT plays important roles in PBMC proliferation [35, 36]. To elucidate whether *4-HM* blocking of NF-*κ*B and NF-AT activation leaded to impairments of PBMC proliferation, effects of *4-HM* on cells proliferation were determined by ^3^H-thymidine uptake. As shown in Figure 9, treatment with PHA for 3 days stimulated cell proliferation as indicated by about 23-fold increase in ^3^H-thymidine uptake. Treatment with the vehicle DMSO (0.1%) affected neither the ^3^H-thymidine uptake in the resting state nor that in the stimulated state. While *4-HM* had little effect on ^3^H-thymidine uptake in resting PBMC, it significantly suppressed the enhanced uptake observable in activated cells (*P* < 0.05, *P* < 0.01, *P* < 0.001). Furthermore, the inhibitory effects of *4-HM* on activated PBMC were concentration dependent. The inhibitory percentages of 2.5, 5, and 10 *μ*M *4-HM* are 17.2 ± 2.7%, 70.7 ± 2.5%, and 95.2 ± 1.3%, respectively, with an 50% inhibitory concentration (IC_50_) of 3.2 ± 1.0 *μ*M. Both NF-*κ*B inhibitor PDTC (20 *μ*M) and NF-AT inhibitor CsA (2 *μ*M) blocked PBMC proliferation induced by PHA. We suggested that deficiency of transcription factors activation such as NF-AT and NF-*κ*B caused impairments of PBMC proliferation induced by PHA.

## 4. Discussion 

In the present study, we observed that *4-HM* isolated from *A. blazei* decreased productions and gene expressions of IL-2, IL-4, IFN-*γ*, and TNF-*α* in activated PBMC. The suppressive actions of *4-HM* were not explained by a drug-induced reduction in cell viability. The activation of NF-AT and NF-*κ*B in the activated cells was attenuated by *4-HM*. The Ca^2+^ mobilization and PKC *θ* activation in PHA-activated PBMC were abrogated by *4-HM*. In addition, results demonstrated that increase in PBMC proliferation induced by PHA was inhibited by *4-HM*. We suggest that *4-HM* interferes with some regulatory events required for PBMC activation and proliferation is suppressed. This is the first report for immunomodulatory functions of *4-HM*.

*4-HM* is a marine steroid and has been isolated from sponges. It has been reported that *4-HM* has antiproliferative effects on several cancer cell lines [37]. The present results showed that *4-HM* suppressed proliferation and cytokine production of human PBMC activated with PHA by modulation of NF-AT and NF-*κ*B activation. It suggests that *4-HM* has immunopharmacological activities. The possible inhibitory effect of DMSO on PBMC was studied in these experiments. DMSO did not change IL-2, IL-4, IFN-*γ*, and TNF-*α* gene expressions, PBMC viability, and cell proliferation. Therefore, the inhibitory function of *4-HM* was not related to DMSO. Results of cell viability tests indicated that there was no significant cell death in PBMC cultures after treatment with 1.25, 2.5, 5, and 10 *μ*M *4-HM* for 4 days. We suggest that under 10 *μ*M and during this time frame, the inhibitory effects of *4-HM* on PBMC were not through direct cytotoxic effects. In the present study, T cells were major proliferating cells in PBMC cultures activated with PHA [9]. Thus, inhibitory effects of *4-HM* on PHA-activated PBMC proliferation could be suggested as suppression on T-cell proliferation. 

The central event in generation of immune responses is the activation and clonal expansion of T cells [9]. Interaction of T cells with PHA initiates a cascade of biochemical events that induces the resting T cells to proliferate [29]. It has been demonstrated in many previous studies with T cells that a series of genes such as IL-2, IL-4, and IFN-*γ* is expressed in a carefully controlled [26, 38]. We demonstrated that production of these cytokines is inhibited by *4-HM*. On the other hand, we also proved that inflammatory cytokine TNF-*α* productions were reduced by* 4-HM*. These results were correlated to Tang et al. findings that extracts from *A. blazei* decreased inflammatory cytokine productions in BALB/c mice [39]. By RT-PCR and real-time PCR, we also proved that *4-HM* decreased IL-2, IL-4, TNF-*α*, and IFN-*γ* mRNA expressions in PBMC stimulated with PHA. We concluded that the impairments of IL-2, IL-4, TNF-*α*, and IFN-*γ* productions were related to *4-HM* suppressing their mRNA transcriptions. 

Two of the earliest T-cell signaling events induced by PHA including NF-AT and NF-*κ*B are required for T cells proliferation and activation [9]. Many previous studies show that NF-AT is an inducible regulatory complex critical for transcriptional induction of many genes in activated T cells, containing IL-2 and IFN-*γ* [13]. Inactivation of NF-AT in mice reduces T-cells number and impairs their proliferation [40]. The transcription factor NF-*κ*B is one of the key regulators of genes involved in the immune response as well as in survival from apoptosis [41]. Further, productions of IFN-*γ* and IL-2 were reduced by inhibition of NF-*κ*B and NF-AT [21, 36, 41]. In the previous study, we also proved that PDTC (NF-*κ*B inhibitor) and CsA (NF-AT inhibitor) reduced IL-2, IFN-*γ*, TNF-*α*, and IL-4 gene expression in PBMC induced by PHA [16]. Both NF-AT and NF-*κ*B are involved in regulation of IL-4 and TNF-*α* genes [42, 43]. In the present study, the luciferase reporter assays were applied to determine inhibitory effects of *4-HM* on NF-AT and NF-*κ*B activation. Furthermore, we also used the immunofluorescence staining and confocal microscopy to confirm the inhibitory effects of *4-HM* on NF-AT and NF-*κ*B activation. The preliminary results showed that NF-AT and NF-*κ*B were predominantly cytoplasmic in resting PBMC, but these signals disappeared upon PHA stimulation and accumulated in the nuclear (fluorescence intensity = 6.8 units/*μ*m^2^). In the presence of *4-HM *(10 *μ*M), NF-AT and NF-*κ*B decreased in the nuclear (fluorescence intensity = 2.2 units/*μ*m^2^) and remained cytoplasmic. It suggested that *4-HM* blocked NF-AT and NF-*κ*B nuclear translocation and activation in PBMC induced by PHA. Thus, the inhibition of *4-HM* on IL-2, IL-4, TNF-*α*, and IFN-*γ* gene transcripts in PBMC induced by PHA could be explained by *4-HM* which inhibited NF-AT and NF-*κ*B activation.

**Figure 10:**
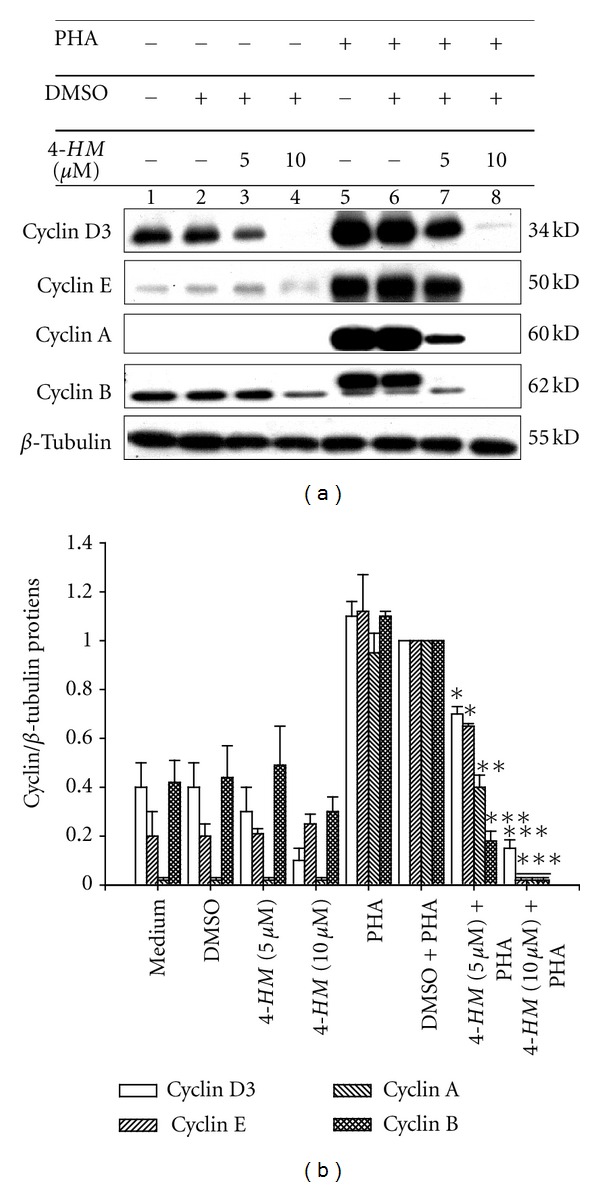
Effects of 4-HM on cyclins protein expression in PBMCs. (a) PBMC (1 × 10^7^ cells) were treated with medium, DMSO (0.1%), or *4-HM* (5, 10 *μ*M) in the presence or absence of PHA (5 *μ*g/mL) for 72 hr. Lysates (50 *μ*g/mL) were collected and run on a 10% SDS-PAGE gel and analyzed by immunoblotting with anti-cyclin D3, cyclin E, cyclin A or cyclin B. (b) The ratio of cyclin to *β*-tubulin was shown in bar graph. Each bar represents mean ± SD from three independent experiments. *P < 0.05, **P < 0.01, ***P < 0.001: versus DMSO + PHA control.

As a consequence of an increase of [Ca^2+^]_i_ levels, calcineurin, a Ca^2+^/calmodulin-dependent protein phosphatase, is activated, leading to dephosphorylation of NF-AT and its subsequent activation and nuclear translocation [44]. We have demonstrated that the magnitude of the Ca^2+^ signal in PBMC triggered by PHA was affected by *4-HM*. We concluded that this could affect activation of NF-AT in PBMC stimulated with PHA. Additionally, activated phospholipase C*γ* (PLC*γ*) triggers activation of PKC *θ* [36], and PKC *θ* activation and membrane translocation sequentially result in the phosphorylation and degradation of I*κ*B [45] and activation of NF-*κ*B in T lymphocytes [46, 47]. It has been proved that PKC *θ* is a key enzyme for regulation of T lymphocytes' survival [48]. By the Western blotting, we proved that *4-HM* decreased PKC *θ* activation in PHA-activated PBMC. We suggested that *4-HM* impairment of NF-*κ*B activation was related to blocking of PKC *θ* activation. Both Ca^2+^ signal and PKC *θ* activation in PBMC were decreased by *4-HM* and these would cause the reduction of IL-2, IL-4, TNF-*α*, and IFN-*γ* gene expressions. Since T lymphocyte proliferation is primarily mediated by IL-2, inhibition of IL-2 production is a central mechanism of action of several immunosuppressants including CsA, which induces arrest activation and proliferation of T cells by inhibiting IL-2 transcription [49]. Production of IL-2, IL-4, and IFN-*γ* is important in coordinating the activation and proliferation of T lymphocytes [12, 26]. Abrogation of NF-*κ*B and NF-AT activity in the T-cell lineage of mice caused a decrease in proliferation of T cells [40, 47]. In the present study, *4-HM* reduced the proliferation in PBMC activated with PHA. It may be expected that the failure to activate NF-*κ*B and NF-AT and produce IL-2, IL-4, and IFN-*γ* are the reasons that PBMCs do not proliferate. Furthermore, cyclin expression plays an important role in eukaryotic cell proliferation [50]. To confirm *4-HM* inhibitory activities on PBMC proliferation, effects of *4-HM* on cyclins expression in PHA-activated PBMC were determined by Western blotting (Figure 10). Compared with vehicle control (DMSO + PHA group), *4-HM* (5, 10 *μ*M) significantly decreased the levels of cyclins D3, E, A, and B proteins in PBMC induced by PHA. We concluded that *4-HM* inhibited PBMC proliferation activated with PHA.

In conclusion, from the present results, we hypothesize that inhibitory mechanisms of *4-HM* on PHA-activated PBMC proliferation, at least in part, are related to (1) decrease of [Ca^2+^]_i_ in the cells; (2) interruption of PKC *θ* activation; (3) reduction of NF-AT and NF-*κ*B activation; (4) blocking of IL-2, IL-4, TNF-*α*, and IFN-*γ* gene expressions and productions; and (5) inhibition of PBMC proliferation. It is believed that arthritis, asthma, cough, and rheumatism are related to overexpression of inflammatory responses [50, 51]. Increased activity of NF-*κ*B transcription factor has been documented in chronic tissue inflammation; accordingly, pharmacologic blockade may become particularly important in the treatment of inflammatory diseases [51, 52]. Both IL-4 and TNF-*α* play important roles in inflammation [52, 53]. It is proved that mice deficiency in PKC *θ* has reduced incidence and severity of inflammatory diseases such as multiple sclerosis and allograft rejection [48]. Thus, results of the present study indicate that *4-HM* included in *A. blazei* may also have acted to reduce tissue inflammation in part by inhibiting PBMC proliferation, cytokine gene expressions such as IFN-*γ*, IL-4, and TNF-*α*, and NF-*κ*B and PKC *θ* activation. Our observation correlated with *A. blazei* putative pharmacological activities [54, 55].

## Figures and Tables

**Table 1:** The primer sequences used for amplification of cytokines and *c-fos* gene in PBMC.

Cytokine	Sequence	Predicted size (bp)
IL-2	5′: GTC ACA AAC AGT GCA CCT AC	262
	3′: GAA AGT GAA TTC TGG GTC CC	
IL-4	5′: CGG CAA CTT TGA CCA CGG ACA CAA GTG CG	449
	3′: AGG ACA CTT CCT TCG GTT GGT CTC ATG CA	
IFN-γ	5′: TCT GCA TCG TTT TGG GTT CTC	290
	3′: CTC TAC TGA AGC TTT TCG ACT	
TNF-α	5′: ACA AGC CTG TAG CCC ATG TT	428
	3′: TCA GAC CCG TCC AGA TGA AA	
*c-fos *	5′: ATC AGC AGC ATG GAG CTG AAG ACC	606
	3′: CGT ACC TCA CAC ATA ACA AGG GTC	
GAPDH	5′: TGA AGG TCG GAG TCA ACG GAT TTG GT	983
	3′: CAT GTG GGC CAT GAG GTC CAC CAC	

**Table 2:** Effects of *4-HM* on cytokine gene expression in PBMC determined by real-time PCR.

	Relative cytokine mRNA levels (% referred to DMSO + PHA group)			
	IL-2	IL-4	IFN-*γ*	TNF-*α*
Medium	5.5 ± 0.8	18.0 ± 0.9	5.0 ± 0.01	15.0 ± 0.05
PHA	118 ± 10	88.0 ± 9.0	110 ± 8.3	105 ± 9.9
DMSO	5.2 ± 0.03	18.0 ± 1.8	4.8 ± 0.03	16.8 ± 0.03
DMSO + PHA	100 ± 0.0	100 ± 0.0	100 ± 0.0	100 ± 0.0
2.5 *μ*M *4-HM *	4.9 ± 0.02	15.0 ± 0.09	4.2 ± 0.005	17.5 ± 0.08
2.5 *μ*M *4-HM* + PHA	35.0 ± 8.0**	36.0 ± 3.4**	39.0 ± 2.5**	51.0 ± 6.8**
5.0 *μ*M *4-HM *	5.0 ± 0.01	6.8 ± 0.09	5.1 ± 0.006	17.0 ± 0.5
5.0 *μ*M *4-HM* + PHA	16.5 ± 6.1**	18.0 ± 3.6**	20.0 ± 8.3**	35.0 ± 5.5**
10 *μ*M *4-HM *	5.0 ± 0.6	5.2 ± 0.04	5.4 ± 0.4	14.0 ± 2.6
10 *μ*M *4-HM* + PHA	13.2 ± 4.5**	12.4 ± 3.3**	12.0 ± 1.9**	23.0 ± 5.2**

PBMC (5 × 10^6^) activated with or without PHA (5 *μ*g/mL) in the presence or absence of *4-HM *(2.5, 5.0, 10 *μ*M) for 18 hr. The total cellular RNA was isolated from PBMC and aliquots of 1 *μ*g of RNA were reverse transcribed for synthesis of cDNA. Briefly, 10 *μ*L of cDNA was applied for the real-time PCR test as described in Section 2. **P < 0.01: versus DMSO + PHA group.

## Abbreviations

PBMC: Peripheral blood mononuclear cells
4-*HM*: 4-hydroxy-17-methylincisterol
PHA: Phytohemagglutinin
ELISA: Enzyme-linked immunosorbent assay
RT-PCR: Reverse transcription-polymerase chain reaction
[Ca^2+^]_i_: Intracellular calcium concentration
IL-2: Interleukin-2
IFN-*γ*: Interferon-*γ*
TNF-*α*: Tumor necrosis factor-*α*
PKC *θ*: Protein kinase C theta
CsA: Cyclosporine A
PDTC: Pyrrolidine dithiocarbamate
EtOH: Ethanol
EtOAc: Ethyl acetate
CH_2_Cl_2_: Dichloromethane
BuOH: Butanol
MeOH: Methanol
DMSO: Dimethylsulfoxide
HBSS: Hanks' buffer saline solution
FCS: Fetal calf serum
DEPC: Diethyl pyrocarbonate
EDTA: Ethylenediaminetetraacetate
DTT: Dithiothreitol.
